# Preconception Counseling in Couples Undergoing Fertility Treatment

**Published:** 2012-06-19

**Authors:** Nafisehsadat Nekuei, Mohammad Hossein Nasr Esfahani, Ashraf Kazemi

**Affiliations:** 1 Department of Midwifery and Reproductive Health, School of Nursing and Midwifery, Isfahan University of Medical Sciences, Isfahan, Iran; 2 Department of Reproduction and Development, Reproductive Biomedicine Research Center, Royan Institute for Animal Biotechnology, ACECR, Isfahan, Iran; 3 Tehran University of Medical Sciences, Tehran, Iran

**Keywords:** Infertility, Preconception, Counseling

## Abstract

**Background:**

The preconception period is crucial to fertility and pregnancy health. Offering education and counseling to couples being treated for infertility improves the outlook of treatment. The aim of this study is to assess preconception education and counseling in a population of Iranian couples treated for infertility.

**Materials and Methods:**

This is a cross-sectional study of 268 individuals who presented to fertility clinics across the city of Isfahan, Iran. Questionnaires and patient records were used to collect data. We evaluated the components of standard preconception counseling (lifestyle, diet, sexual health, substance abuse, and social counseling) versus preconception counseling offered to couples that were receiving infertility treatment (failure, follow-up, and side effects of treatment).

**Results:**

We found that no counseling had been given to about 76.9% about lifestyle, 70.9% about diet, 90.7% about sexual health, and 90.7% about the psychosocial aspects of infertility. No counseling had been given to 46.6% of individuals about a follow-up and also to 46.6% of individuals about the side effects of treatment. In more than 75% of the cases, counseling was offered to couples whose etiology of infertility was unknown.

**Conclusion:**

We have found serious flaws in the education and preconception counseling of infertile Iranian couples; action is required by medical and health teams to address these shortcomings.

## Introduction

Preconception counseling can improve maternalfetal health, both during pregnancy and afterwards. Due to limitations of perinatal care and the importance of maternal health before pregnancy, counseling should be offered before conception. Maintaining a healthy environment around the oocyte and the embryo is essential to the health of the child. Preconception counseling helps prepare for pregnancy and secures the health of the oocyte/ fetus. Preconception training and counseling prepares the parents for a healthy pregnancy, creating ideal conditions for the oocyte and ultimately the fetus. Infertile couples may have been exposed to conditions that contributed to their fertility.

The same factors can affect the health and outcome of pregnancy. Fertility treatment may be lengthy, and failure is not uncommon. Their presence at treatment centers prior to conception and their high motivation to achieve a healthy pregnancy offer a good opportunity for preconception training and counseling, possibly leading to an improved outcome of pregnancy. Despite planning vigorously to overcome infertility, couples may lose sight of critical preconception care, which is the key to a successful pregnancy (1-3). Several studies have demonstrated that preconception education/counseling can help identify and remove fertility risk factors by improving lifestyle and enhancing the follicular microenvironment and sperm function (4-7). Lifestyle, diet, sexual health, substance abuse, and psychosocial factors influence not only the outcome of fertility treatment, but also pregnancy health, which is central to preconception care (8-10). Couples presenting to fertility clinics are highly motivated to achieve pregnancy; this offers a rare opportunity to offer preconception education/counseling and improve their chances of success (11). This study aims to assess preconception education/counseling in couples treated for infertility.

## Materials and Methods

This is a quantitative, descriptive, cross-sectional study of 268 individuals who received treatment for infertility for at least the second time at specialist clinics across the city of Isfahan, Iran. The study was conducted between September 2008 and May 2009. Excluded from the study were couples who used donated eggs/fetuses or gestational surrogacy techniques. Simple sampling method was used. Data were collected from questionnaires, interviews and patient medical records after obtaining written informed consent from subjects.

The research tool consisted of a two-part questionnaire: personal details were entered in part one and included ten items (age, education level, occupation, infertility category, infertility cause, infertility duration, treatment duration, treatment done, outcome of previous treatment, and treatment times). Part two obtained information about the couples’ preconception education/counseling as outlined in reference textbooks. It included two sections: a. personal counseling: life style (exercise, rest, genetic counseling, and personal health), diet (diet and weight balance) sexual health (sexual activity and sexual transmitted infections), substance abuse (addiction, smoking, and drug use) and psychosocial factors; b. treatment counseling: failure, follow up, and side effects.

This section was compiled using specialist textbooks in the field of preconception counseling. Data collection was conducted using medical records or by interviews in cases where records were incomplete. The counseling received by couples was described as 'complete', 'incomplete', or 'not given'.

A pilot study was performed on 20 individuals who were similar to the main study subjects with the intent to assess the reliability of our questionnaire. An alpha reliability coefficient of 0.75 was achieved. Pilot study subjects were not included in the main study. After the completion of sampling, we applied descriptive-analytical statistical methods (Pearson’s chi-square and Spearman correlation analysis) to process data. SPSS software (version 16) was used. The Ethics Committee of the Isfahan University of Medical Sciences approved this study.

## Results

We studied 268 couples who presented to fertility clinics. Ten couples were excluded because their records were incomplete and five others were excluded for personal reasons. The demographic characteristics of subjects are shown in table 1.

**Table 1:** Basic characteristics according to gender


Gender	Females	Males
Basic individualcharacteristics		

**Age (%)**		
< 30 years	150 (56)	69(25.7)
30-35 years	84 (31.3)	117 (43.7)
>35 years	33 (12.3)	82(30.6)
Mean ± SD	29.41 ± 4.99	32.68 ± 5.17
**Education level (%)**		
Less than high school	84 (31.4)	138 (51.5)
High school diploma	115 (42.9)	95(35.41)
University degree	69 (25.7)	30(11.19)
**Occupation (%)**		
House keeper	212 (79.1)	0
Employed	50 (18.7)	75(28)
Self-employed	0	169 (63.1)
Others	6 (2.2)	24(9)


Descriptive analytical methods were used for analysis. We demonstrated that primary infertility was 88.1%, with maximum prevalence related to male factors. In 72.8% were not pregnancy before and the maximum prevalence for the previous treatment was induction ovulation (27.2%). Most couples were undergoing their second cycle of infertility treatment (44.8%), and 69% failed the previous treatment. The mean infertility duration was 5.06 (years) and the duration of infertility treatment in couples was 3.64 years. We found that 76.9% of couples had no lifestyle counseling, 70.9% had no diet counseling, 90.7% no sexual health counseling, 90.7% no counseling on the psychosocial aspects of infertility. 56.34% received incomplete counseling on substance abuse, 67.9% received complete counseling on treatment failure. 46.6% of couples received no counseling on follow-up procedures, and 46.6% received no counseling on the side effects of treatment.

Most counseling cases were concerned with treatment failure (67.9%), with the fewest cases being related to sexual health (0.8%). Table 2 shows the relationship between causes of infertility and counseling in different areas. Statistical analysis using descriptive methods demonstrated that complete counseling was most offered most frequently in cases where the origin of infertility was unknown.

The chi square test was used to examine the relationship between counseling and infertility causes. The results revealed a significant relationship between causes of infertility and individual (p=0.02) as well as therapeutic counseling (p=0.01).

Analysis of the correlation of educational components/preconception counseling with some individual characteristics seen in couples is shown in table 3. Spearman, Pearson’s, and chi-square coefficient, respectively were used to assess the correlations between educational level, treatment times, durations of treatment and infertility with preconception counseling items.There was a direct relation seen in some items. According to table 3 there is a direct correlation between education and sexual health, substance abuse, treatment failure, and treatment complications.

**Table 2:** Frequency distribution of preconception counseling according etiology of infertility


Counseling items	Treatment (offered)			Basic items (offered)		

**Etiology of infertility**	>75%	50-75%	<50%	>75%	50-75%	<50%
**Ovarian factor**	27	14	39	1	2	76
	34.2%	17.7%	48.1%	1.3%	2.5%	96.2%
**Pelvic causes**	26	6	22	0	4	50
	48.1%	11.1%	40.7%	0%	7.4%	92.6%
**Male causes**	31	25	48	0	4	100
	29.8%	24%	46.2%	0%	3.8%	96.2%
**Unknown causes**	19	6	6	3	3	25
	61.3%	19.4%	19.4%	9.7%	9.7%	80/64%


**Table 3:** Correlation between counseling and basic individual characteristics


Counseling items	Individual		Characteristics	
	Education	Treatment times	Duration of infertility treatment	Duration of infertility

**Life style**	0.06	- 0.02	- 0.03	- 0.03
**Nutrition**	0.06	0.07	0.12	0.11
**Sexual health**	0.12*	0.11	0.03	0.73
**Substance abuse**	0.13*	0.27**	0.2*	0.18*
**Psychosocial counseling**	- 0.06	0.09	0.15*	0.23**
**Treatment failure**	0.01*	0.15*	0.12	0.09
**Treatment follow-up**	0.06	0.4**	0.33**	0.33**
**Treatment complications**	0.13*	0.27**	0.26**	0.27**


* p<0.05, ** p<0.001 and Others: p>0.05.

## Discussion

In this study we have assessed the availability of preconception education/counseling to infertile couples. Researchers recommend that training and counseling related to various medical, psychological, social, and health-related aspects of infertility be integrated into routine programs for infertility treatment (12). Counseling links the various components of the infertility treatment cycle and helps obviate the possible causes of infertility, which may not even be the culprit in a given. Moreover, counseling fills the possible gaps in the treatment process, ultimately contributing to its improvement. Studies have shown that infertile women may not be adequately informed about various aspects of infertility and are willing to be offered counseling (3).

Based on our review of the literature, this is probably the first study of its kind to comprehensively address the various aspects of preconception education/counseling offered to infertile couples. The demographic characteristics of individuals in this study make them a representative sample of infertile Iranian couples, because the distribution of infertility-related variables/indicators in our study is similar to that in other studies (13, 14).

This study demonstrates significant flaws in all aspects of counseling in basic areas such as lifestyle, diet, sexual health, substance abuse, and psychosocial aspects of infertility. Other studies have shown that factors such as obesity, poor diet, and substance abuse influence fertility, the outcome of infertility treatment, perinatal complications, and the outcome of pregnancy (15-26). Some studies have recommended preconception screening (27).

According to one study, most infertility specialists recommend cessation of smoking (28). Some infertile women think they should avoid physical activity during pregnancy (29). The flaws in the current system for provision of preconception education/counseling to infertile couples in Iran need addressing.

We have found that most couples had not received any sexual counseling. Studies have demonstrated that sexual disorders are prevalent among infertile couples, possibly contributing to their infertility (30-32); better sexual health counseling is therefore warranted.

The findings of the present study may have been influenced by either Iranian cultural behaviors (which tend to discourage open conversation about sexual matters), the counselors’ tendency to consider the couples’ sex lives as their private matters, or failure in appreciating the significance of a healthy sexual relationship in achieving a successful pregnancy.

Most individuals in our study had not received any psychosocial counseling. Several studies have shown that couples treated for infertility experience a high level of anxiety (33, 34).

Mental stress can lead to infertility, reduced success of *in vitro* fertilization, and poor pregnancy outcome (35-38). Infertile individuals require psychological support (12, 38), given the large variety of psychological disorders among infertile individuals, the reciprocal influence of such disorders and fertility, and the effectiveness of counseling in reducing stress and improving treatment, it seems essential to offer preconception psychosocial counseling at infertility clinics (12, 39-41). Psychosocial counseling needs to be integrated into medical treatment, not only because counseling provides vital emotional support, but also because it can contribute towards reducing the drop-out rate in treatment (42).

Another study has shown a wide range of psychological disorders to be more prevalent among infertile couples compared to healthy ones (43). Defects in psycho-social counseling observed in our study can be attributed to the therapists’ lack of awareness, specialist training, and well informed attitudes in the field. Furthermore, psychosocial counseling requires adequate time, the establishment of an effective rapport between therapists and patients, and holistic appreciation of the patients’ conditions. This is partly due to high demand on service providers and inadequate personnel.

A direct significant relationship that was seen between the causes of infertility and the frequency of personal counseling was probably related to the fact that infertility treatment providers were more likely to offer counseling to individuals with idiopathic infertility. This may help tackle the hidden causes of infertility. Personal preference for counseling may also have played a role.

We found that the majority of individuals had received complete counseling about the failure and side effects of treatment, while no counseling had been offered about follow-up. It is crucial to keep the infertile couples informed about the course and implications of treatment.

Awareness of failure rates, possible side effects, and treatment follow-up influences the outcome and cost of infertility treatment (44, 45). Counseling relieves anxiety and facilitates adaptation with infertility and treatment (41, 46). Infertility counseling and complications of treatment are often the focus of attention by both therapists and couples, thus related counseling has been routinely offered. Follow-up procedures and information may not be offered due to the therapist’s haste to end the interviews in time for the next session; this can significantly undermine the treatment process. Inadequate counseling (<50% of standard) in a high percentage of subjects reflects general weaknesses in counseling infertile couples. The etiology of infertility does not result in better counseling. In general, the weakness of the treatment system, inadequate resources, bad timing, inadequately qualified/ inefficient personnel, and uninformed clients are among the factors that contribute to less than adequate counseling.

We found a significant direct relationship between the etiology of infertility and amount of treatment-related counseling. More counseling was offered when the cause of infertility was unknown; these may be accounted for by the patients’ greater concern about their condition, which resulted in more frequent visits. A lower amount of counseling when a male etiology was suspected was probably due to men’s lower involvement in the treatment process, lower attention/sensitivity to the problem, and forgetfulness. This may in part be related to the Iranian male’s cultural tendency to consider their partners as the source of infertility early in the treatment. Some men find it hard to accept that the problem may in fact have a male cause; they remain in denial and discontinue, or fail to attend counseling. Counseling offered by a male counselor, educating the clients about fertility, and improved education will probably increase the demand for counseling in this group of patients.

We have observed a direct relationship between education and some individual characteristics of the patients. Higher education apparently made couples more likely to seek counseling; individual attitudes, personal reactions to suggestions, and the quality of encounters with the therapy team possibly played a role. In other instances no relationship was observed. It was possible the infertility treatment team assumed that well educated couples did not require as much counseling as those less educated. Although educated couples may have some health information, this may be inadequate and complete preconception counseling should be provided. Certain elements in counseling which have exhibited a direct relationship with the number of treatments, length of treatment, and length of infertility may have done so by increasing the sensitivity/interest of both the couples and therapists.

The infertility treatment process usually begins with the induction of ovulation. At this stage, this may still be considered a routine treatment without proper counseling and emphasis on infertility. Thus, in this study the amount of counseling increased with repeated visits and increased duration of treatment. Our results were reasonably consistent with the current status of the treatment system.

To improve the outcome of infertility treatment, we propose that any factor which can shorten the duration of treatment and increase success rates (including counseling) be considered from the outset of treatment. Where significant relationships were not seen, other factors may have been involved.

Further studies are warranted to find stronger links and causative factors.

## Conclusion

The provision of preconception counseling to infertile couples in Iran is largely flawed. The greater than usual importance of achieving a successful pregnancy in this group of patients warrants special attention to preconception counseling. We propose that providers and recipients of infertility treatment be sensitized about the importance of counseling; this can be accomplished by using standard forms and educational pamphlets/brochures, as well as virtual training.
